# The Effects of Shilajit on Brain Edema, Intracranial Pressure and Neurologic Outcomes following the Traumatic Brain Injury in Rat

**Published:** 2013-07

**Authors:** Mohammad Khaksari, Reza Mahmmodi, Nader Shahrokhi, Mohammad Shabani, Siavash Joukar, Mobin Aqapour

**Affiliations:** 1 Physiology Research Center, Kerman University of Medical Sciences, Kerman, Iran; 2 Jiroft Education, Jiroft, Iran; 3 Neuroscience Research Center ,Kerman University of Medical Sciences, Kerman, Iran; 4 Mobin kahroba kimia Company, Kerman, Iran

**Keywords:** Brain edema, Intracranial pressure, Rat, Shilajit, Trauma

## Abstract

***Objective(s):*** Brain edema is one of the most serious causes of death within the first few days after trauma brain injury (TBI). In this study we have investigated the role of Shilajit on brain edema, blood-brain barrier (BBB) permeability, intracranial pressure (ICP) and neurologic outcomes following brain trauma.

***Materials and Methods:*** Diffuse traumatic brain trauma was induced in rats by drop of a 250 g weight from a 2 m high (Marmarou’s methods). Animals were randomly divided into 5 groups including sham, TBI, TBI-vehicle, TBI-Shi150 group and TBI-Shi250 group. Rats were undergone intraperitoneal injection of Shilajit and vehicle at 1, 24, 48 and 72 hr after trauma. Brain water content, BBB permeability, ICP and neurologic outcomes were finally measured.

***Results:*** Brain water and Evans blue dye contents showed significant decrease in Shilajit-treated groups compared to the TBI-vehicle and TBI groups. Intracranial pressure at 24, 48 and 72 hr after trauma had significant reduction in Shilajit-treated groups as compared to TBI-vehicle and TBI groups (*P*<0.001). The rate of neurologic outcomes improvement at 4, 24, 48 and 72 hr after trauma showed significant increase in Shilajit-treated groups in comparison to theTBI- vehicle and TBI groups (*P* <0.001).

***Conclusion:*** The present results indicated that Shilajit may cause in improvement of neurologic outcomes through decreasing brain edema, disrupting of BBB, and ICP after the TBI.

## Introduction

Mortality or permanent disability following the brain injury is a major health challenge throughout the world. Studies show that approximately 1.5-2 millions traumatic brain injuries per year in the United States (1) imposing 10 billion dollars of expense (2). Furthermore, in Iran, there are 210000 accidents per year leading to the 140,000 deaths and 70,000 injured. Therefore, approximately 50-60% of deaths are due to brain injury (3). Although as a result of more improved safety measures and emergency cares, the number of victims has been significantly reduced, the injured cases suffer from significant neurologic problems (2). It is well understood that following TBI, massive ionic influx is facilitated followed by a release of excitatory amino acids such as glutamate (4). It is the most important mechanism of injury following TBI and eventually neuron deficits. At the cellular level, TBI is resulted in cytoskeleton injury and axonal disconnection. Microdialysis experiments in humans have shown the high concentration of extracellular glutamate after brain injury that may cause the injury of adjacent cells, activation of cell death cascade and serial release of excitotoxic destructive molecules (4).

Brain edema and the accompanying raise of intracranial pressure (ICP) are among immediate outcomes of TBI leading to very early death. An essential part of brain edema following TBI is astrocytes edema (cytotoxic edema) (5). Moreover, systemic inflammatory response syndrome (SIRS) and hypotension in TBI may also be consequences of trauma (6). Mortality in brain trauma injury is typically due to the brain edema and the related inflammatory responses rather than ischemia or bleeding (7). For this reason, several studies in relation to the decreasing brain edema have been performed so far, but none has led to the discovery of a definite effective therapeutic approach (8).

In the developed countries, there are several lines of studies about Shilajit and its importance in the treatment of different diseases by health experts and pharmacological organizations. Some have reported the beneficial effects of Shilajit in the treatment of peptic ulcer (9-10), bone pains and fractures (11-12). Asian Shilajit (Mumiju) includes 20% minerals, 15% protein, 5% lipids, 5% steroids and also some carbohydrates, alkaloids and amino acids (13-14). Few therapeutic effects of this substance are as follows: memory improving, neuroprotective, anti-inflammatory and anti-oxidant roles (10, 15-19). The biological effect of Shilajit has been attributed to di-benzo-alpha-pyrone, humic acid and folic acid contents (19-20).

With regard to the several beneficial effects mentioned for Shilajit, we hypothesized that the administration of this substance can be effective in the improvement of post-traumatic injuries. Therefore, in the present study in order to investigate the neuroprotective effect of this substance brain edema, neurological outcomes, BBB permeability, and ICP following the traumatic brain injury.

## Materials and Methods

In this study 105 male N-MARI Albino rats weighing 250-300 g were used. Animals were purchased and kept in the animal room of Kerman School of Medicine at 20-22°C and 12 hr light-dark cycle. Rats had free access to food and water. This research was carried out under the approval of the Medical Ethics committee of Kerman University of Medical Sciences (reference No.EC/KNR/89-13).

***Experimental groups***

Animals were divided into 5 groups each including 3 subgroups (n=7):

1. Sham group: Animals that underwent preparation procedure of inducing trauma but were not exposed to trauma.
2. TBI group: Animals that underwent brain trauma but did not receive any drug.
3. TBI- vehicle group: Animals that received intraperitoneal injection of Shilajit vehicle (normal saline, 0.5 mL/rat) after exposure to TBI.
4. TBI-Shi150 group : Animals that received intraperitoneal injection of 150 mg/kg Shilajit at 1, 24, 48 and 72 hr after trauma(21) .
5. TBI-Shi250 group: Animals that received intraperitoneal injection of 250 mg/kg Shilajit at 1, 24, 48 and 72 hr after trauma.

***Shilajit preparation***

Shilajit was prepared from the local residents of Sardoiyeh in Jiroft/Kerman/Iran. After at least 2-3 times washing, it was dried, powdered and dissolved in normal saline (to obtain a concentration equal to what being used by local residents). Then, it was placed on shaker for 24 hr, centrifuged (5000 g; 10 min) and sterilized in the autoclave. The prepared powder was finally dissolved in normal saline in order to be injected at 150, and 250 mg/kg doses (21).

***Induction of brain trauma***

Tracheal intubation was performed in anesthetized rats for all animals before TBI followed by the exposure to diffuse traumatic brain trauma using an instrument made in Physiology Department of Kerman Afzalipour School of Medicine with Marmarou’s methods (22-23). As it is instructed in this method, a 250 g weight was dropped through a free-falling tube onto the head of anesthetized animal (by halothane in mixture of 70% N_2_O and 30% O_2_) from a 2-m height while a steel disc was attached to the animal’s skull. After brain trauma induction, the animal was immediately connected to the animal respiratory pump (TSA compact, Germany) and as soon as spontaneous breathing it was disconnected from ventilator and returned to the cage to be cared (24).

***Determination of brain water content***

Brain edema was determined by measuring brain water content at the end of 72 hr following trauma, the animal’s brain was removed under irreversible anesthesia and weighed (wet tissue weight). Then, it was placed inside an autoclave (Memmert, Germany) at 60°C for 72 hr to obtain dry tissue. Then it was reweighed and brain water content was calculated using the following formula (25):

Brain water content (%) = [(wet tissue weight-dry tissue weight)/wet tissue weight] ×100

***Determination of blood - brain –barrier permeability***

Blood-brain-barrier (BBB) permeability was determined through measuring extravascular Evans blue dye and using spectrophotometer device. Five hours after trauma, brain vascular permeability was measured by the injection of Evans blue dye via tail vein (26); at 4 hr after trauma, under anesthesia by 50 mg/kg thiopental, 20 mg/kg Evans blue dye 2% (1 ml/kg) was injected through tail vein. One hour after injection (at 5 hr after trauma), thorax was opened and descending aorta was clipped. Then, 200-300 ml isotonic saline solution was infused into the left ventricle for 20 min to remove intravascular Evans blue dye. For this purpose, jugular vein was cut bilaterally and infusion was continued until complete removal of Evans blue (26). Next, the brain was immediately removed and weighed, followed by the homogenization. Then, it was placed in 20 ml solution containing 6 ml sodium sulfate 1 % + 14 ml acetone and then on shaker for 24 hr. In the next step, 1 ml of the supernatant liquid was taken and mixed with 1ml trichloroacetic acid. After being kept in a cold place for 2-3 min it was centrifuged at 2000 cycle/min for 10 min, then 1 ml of the supernatant liquid was taken and the absorbance of Evans blue was measured at 620 nm by spectrophotometer (Pharmacia biotech, Germany). The amount of color based on µg/ mg brain tissue was calculated by the following formula: 

Evans blue dye (µg) in brain tissue (g)= (13.24×20×absorbance)/tissue weight

Higher amount of dye in brain tissue represents more vascular permeability and more severe blood-brain barrier disruption (26).

***Intracranial pressure assessment***

The anesthetized animal (by halothane in mixture of 70% N_2_O and 30% O_2_) was placed in a streotax device made in Physiology Department of Kerman School of Medicine and Kimia Mobin Company for measuring ICP. The area between two ears on the scalp was shaved and cisterna magna area was marked. Intracranial pressures at one hour prior to the trauma and at 1, 4, 24, 48 and 72 hr after trauma were measured by a needle (No.23, depth: 5 mm) connected to the E50 tube of ICP monitoring device (25, 27, 28) by a computer via a coupler using Lab Tutor4.0 software(ADI instrument. Australia).

***Evaluation of neurologic outcomes***

Neurologic outcome was assessed based on (Veterinary Coma Scale) VCS (25, 29) in which the total score (3-15) is obtained by adding the scores of motor response (1-8), visual response (1-4) and respiratory response (1-4)(Table 1) (28). Higher score represents better neurological outcome. In the present study, neurologic outcomes were also assessed at -1, 1, 4, 24, 48 and 72 hr post trauma injury. 

**Table 1:** Veterinary coma scale

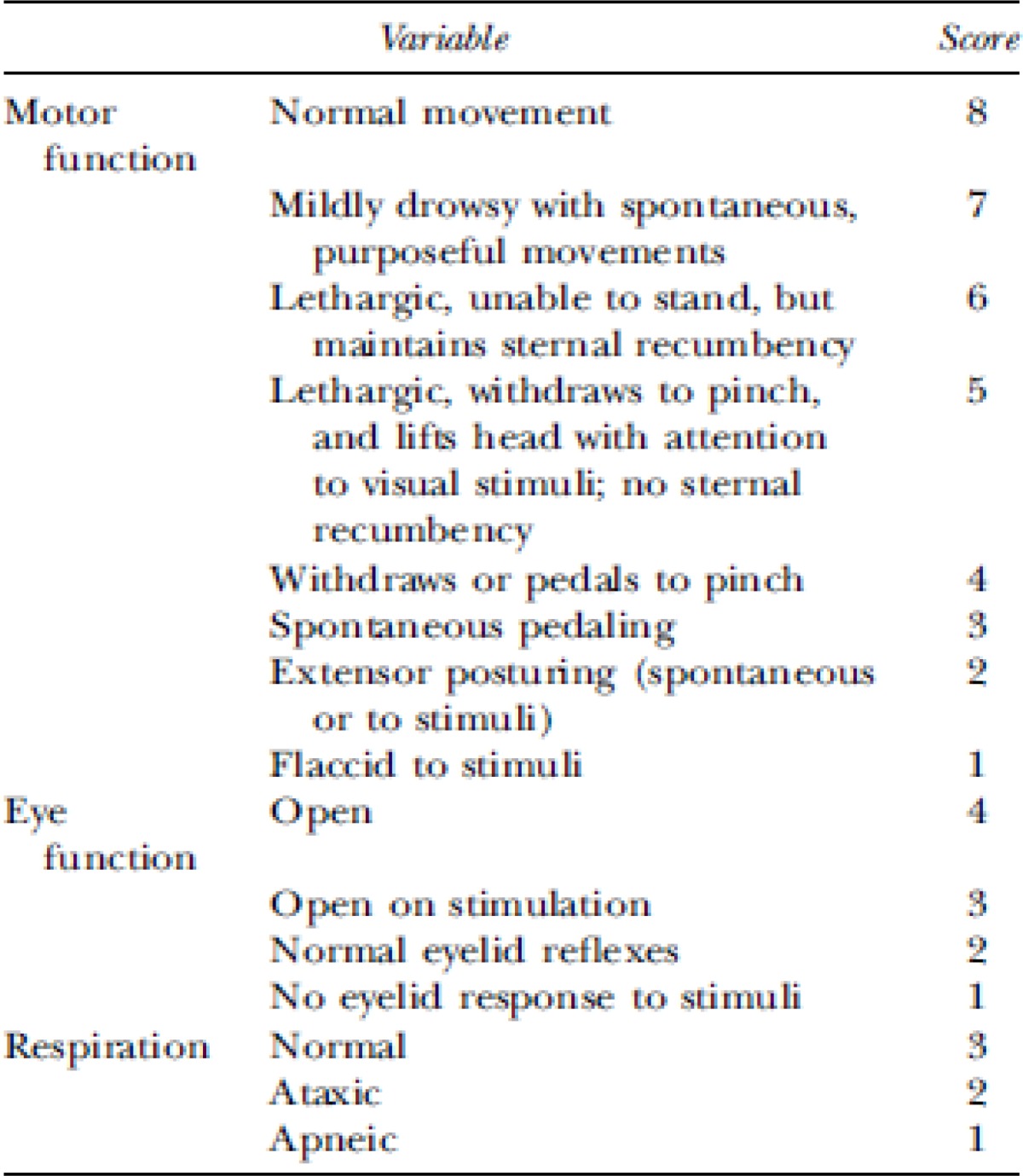

***Statistical analysis***

Repeated measure test was used to detect the interactions among measurement times, neurological scores and groups (*P*<0.001 with Greenhouse-Geisser correction). Following that, ANOVA was used for comparison of mean scores in different groups at -1, 1, 4, 24, 48 and 72 hr after trauma. In the case of finding significant difference in the primary test, Tukey test was applied to determine the differences among groups. All data are presented as mean ±SEM and *P*<0.05 was considered as least statistical significant level.

## Results

**Figure 1:**
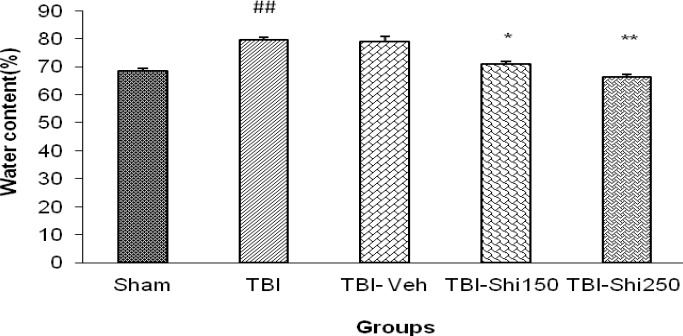
The effect of different doses of Shilajit on brain water content.

The effects of Shilajit on brain water content:

Figure 1 shows the brain water content in TBI group (79.53±1.05) with the significant increase in comparison to the sham group (68.41±0.87) (*P*<0.01). There was no significant difference among TBI and TBI- vehicle groups. Furthermore, in the TBI-Shi150 (70.80%±0.915) and TBI-Shi250 groups (66.28%±0.98) it was shown that there is a significant decrease compared to the TBI-vehicle (78.83%±1.95) and TBI group (*P*<0.05 and *P*<0.01 respectively). 

Data are presented as mean ±SEM. (n= 7) *P<0.05: significant difference of TBI-Shi150 -treated group with TBI-Veh -treated and TBI groups.

** P <0.01: significant difference of TBI-Shi150 -treated group with TBI-Veh -treated and TBI groups.The also significant difference of TBI group with sham group, ## *P* <0.01: significant difference of TBI group with sham group

**Figure 2:**
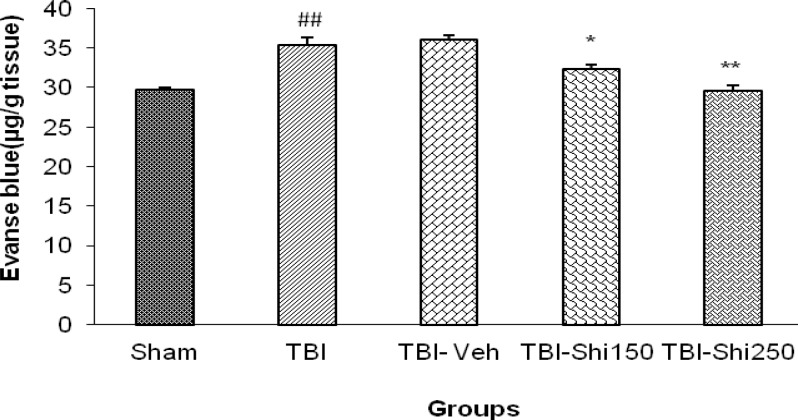
The effect of different doses of Shilajit on brain Evans blue dye content.

Data are presented as mean ±SEM. (n= 7) *P<0.05: significant difference of TBI-Shi150 group with TBI-Veh and TBI groups. **P<0.01: significant difference of TBI-Shi250 group with TBI-Veh and TBI groups, ## *P*<0.01: significant difference of TBI group with sham group.

***The effects of Shilajit on Evans blue dye content***

Comparison of the amount of Evans blue dye in the studied groups has been shown in Figure 2. The amount of Evans blue dye in TBI group (35.4 ± 0.89) showed significant increase in comparison to the sham group (29.7 ±0.32 µg/g tissue) (*P*<0.01), while there was no significant difference among TBI and TBI- vehicle groups. This amount in TBI-Shi150 - group (32.3 ±0.32 µg/g tissue) and TBI-Shi250 - group (29.5±0.72 µg/g tissue) in comparison to those in TBI-vehicle- group (36.1±0.54 µg/g tissue) and TBI group, showed significant decrease (*P*<0.05 and *P*<0. 01 respectively). 

**Figure 3:**
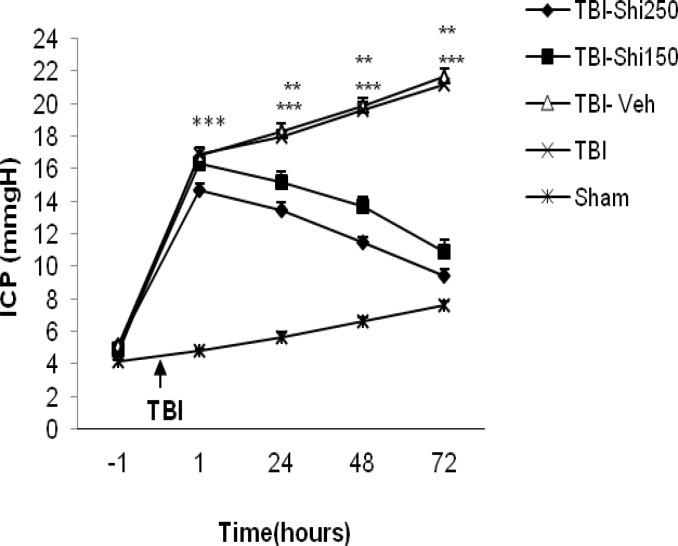
The effect of different doses of Shilajit on intracranial pressure ICP.

Data are presented as mean ±SEM. (n= 7) **P<0.01: significant difference of TBI-Shi150 group with TBI-Veh- and TBI groups at 48 and 72 hr after TBI and with sham group at 1, 24 and 48 hr after TBI. ***P<0.001: significant difference of TBI-Shi250 group with TBI-Veh and TBI groups at 48 and 72 hr after TBI and with sham group at 1, 24 and 48 hr after TBI

**Figure 4:**
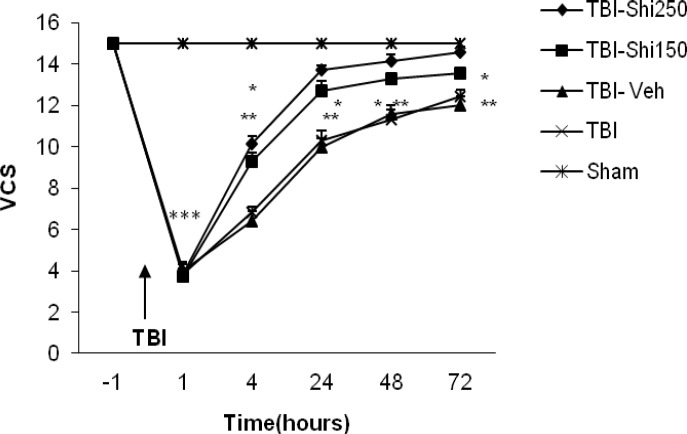
The effect of different doses of Shilajit on (Veterinary Coma Scale )VCS .

Data are presented as mean ±SEM. (n= 7) *P<0.05: significant difference of TBI-Shi150 group with TBI-Veh and TBI groups at 4, 24,48 and 72 hr after TBI and with sham group at 1, 24 and 48 hr after TBI. **P<0.01: significant difference of TBI-Shi250 group with TBI-Veh and TBI groups at 48 and 72 hr after TBI and with sham group at 4, 24 and 48 hr after TBI. ***P<0.001: significant difference of sham group at 1 and 4 hr after TBI with other groups

***The effects of Shilajit on intracranial pressure***

Figure 3 shows amount of intracranial pressure in all groups. Before trauma induction, there was no significant difference in the amount of intracranial pressure among groups, while there was significant difference among sham and TBI groups in all hours after trauma. However, ICP showed significant decrease in TBI-Shi 250 group at post-trauma hours of 24 (13.43±0.485 mm/Hg), 48 (11.43±0.365 mm/Hg) and 72 (9.43±0.365 mm/Hg) and in TBI-Shi 150 group at the same hours (15.14±0.65, 13.71±0.56 and 10.89±0.71 respectively). The values were compared with the values of the TBI-vehicle group (18.29±0.48, 19.86±0.497 and 21.7±0.45 respectively, *P*<0.001) and TBI group (18.0±0.375, 19.75±0.53 and 21.7±0.45 respectively, *P*<0.01). 

***The effects of Shilajit on brain neurologic outcomes***

The mean scores of brain neurologic outcomes in different groups are shown in Figure 4. The score of neurologic outcomes in TBI-Shi 250 group at post-TBI hours of 4, 24, 48 and 72 and at the same hours in TBI-Shi 150 group showed significant increase in comparison to the values of TBI-vehicle (*P* <0.01) and TBI groups, *P* <0.05). When the score of neurologic outcomes were evaluated, there was a significant decrease between sham and TBI groups in all hours after TBI (legend have not shown).

## Discussion

Brain edema is the result of increase in brain tissue water content associated with blood-brain barrier disruption together with the filtration of intracellular and interstitial inflammatory factors (30). Among causes of brain edema, Na+/K+ pumps disruption following energy loss due to brain ischemia, increase of capillary filtration coefficient, increase of Na+ and Ca++ influx could be mentioned. These may cause due to the elevated glutamate release and disturbance of matrix metalloproteinase (MMP) expression (31-32). In the present study, it was recognized that low and high doses of Shilajit will decrease the brain water content by 7.5% and 13.8% respectively. According to the recent reports (10), Shilajit has significant anti-inflammatory effects in all three types of acute, sub-acute and chronic inflammation and can remove free radicals. There are some evidence showing the effects of Shilajit on increasing superoxide dismutase (SOD), catalase (CAT) and glutathione peroxidase (GPX) activities in corpora striatum and frontal cortex of rats (18). Shilajit can significantly decrease carrageenan-induced edema in rat paw (33). In some studies, it has also been reported that Shilajit has anti-allergic effects on histamine release and causes mast cells degranulation (34). All of these points can justify the results of the present study.

In another part of this study, low and high doses of Shilajit could respectively decrease Evans blue dye content of brain by 10.5% and 16%. In other words, Shilajit could reduce the permeability of blood-brain barrier. These effects may be related to the anti-inflammatory and neuroprotective effects of Shilajit (10, 13). Few studies have reported the brain tissue damage due to inflammatory damage of glial cells, microvascular damage, excitotoxicity and aberrant ionic homeostasis in neurons (35). Damage of central nervous system causes neuroinflammatory responses including microglia and astrocytes activity (36-38), blood-brain barrier permeability reduce (39) , ICP increase, CPP decrease (25) and acute increase of proinflammatory cytokines such as TNF-α, IL-1β and IL-6 (40). Anti-edema effects of Shilajit could be related to its antioxidant and anti-inflammatory effects (19).

The present study has also showed that different doses of Shilajit can be efficient in decreasing post-TBI intracranial pressure in a way that immediately after TBI, ICP in TBI-vehicle and TBI groups was significantly higher compared to that in the sham group. Raise of ICP occurred about one hour after trauma continued for 72 hr. Sham group animals, such as TBI group animals, showed a relative increase of ICP at different post-traumatic hours. The reason of ICP elevation in sham group is not clear; however, inserting the probe of ICP assessment device could be an explanation for ICP increase in all groups (41) . Probable causes of ICP increase in TBI group are increase of brain blood volume or constriction of menengial layers surrounding the brain (42), hypoxia (43), cerebral blood flow decline(44). Different doses of Shilajit have decreased the ICP and increased CCP (results have not been presented) in compared to TBI-vehicle animals at 24, 48 and 72 hr after trauma. One of the standard cares in severe TBI is ICP control and therapeutic approaches causing improvement after TBI, have been designed based on maintaining CPP at normal level. According to the previous studies, Shilajit may prevent post-traumatic raise of ICP for different reasons. Because of containing bioactive di-benzo-alpha-pyrone associated with humic acid and fulvic acid, Shilajit act as carrier molecules for main components and may play an important role in decreasing vascular damages accompanied with oxidative stress (45). It has been also reported that Shilajit maintain the required oxygen level of body during hypoxia through increasing blood’s oxygen transfer capacity and improving circulation in tissues (46-47). Another mechanism reported for anti-edema effect of Shilajit. Therefore, this substance as a diuretic factor, can remove excessive liquids from the body (34, 47). All these can explain decreasing effects of Shilajit on post-trauma ICP.

Neurologic outcomes assessment showed higher scores in Shilajit-treated groups at different post-trauma hours compared to TBI-vehicle-treated and TBI groups. This finding shows that Shilajit is efficient in restoring neurologic behaviors from early hours after brain trauma. This increase in neurologic score can be through ICP decline and CPP increase (29, 48-49) . It has been reported that low neurologic scores are related to vascular spasm and deficiency of cerebral perfusion (50). Low cerebral perfusion after TBI is associated with reduction in tissue Oxygen (51) . Some other studies have shown that ShilajitShilajit maintain the required Oxygen level of body during hypoxia through increasing blood’s Oxygen transfer capacity and improving circulation in tissues (46). Therefore, it is probable that Shilajit increases neurologic scores and speeds up behavioral recovery after TBI through increasing cerebral blood flow and decreasing ICP.

## Conclusion

Overall, the results of present study showed that Shilajit administration has neuroprotective effects in post-traumatic injuries through decreasing brain edema, blood-brain barrier permeability and ICP. Therefore, may be through these mechanisms post-trauma neurologic score will be improved, however; further studies are required to determine other functional mechanisms of Shilajit.
